# Analysis of infection characteristics and clinical predictive indicators of different bacterial diabetic foot in Yunnan area

**DOI:** 10.3389/fendo.2026.1826314

**Published:** 2026-05-08

**Authors:** Rong Zhu, Keqiang Mei, Zehui Liu, Lijuan Ma, Shiyu Feng, Yaping Zhao, Lixin Chen, Zizhou Wang, Zhenqin Ran, Rong Yang, Rui Han

**Affiliations:** Department of International Medicine, The First Affiliated Hospital of Kunming Medical University, Kunming, Yunnan, China

**Keywords:** antibiotics, bacterial infection, diabetic foot, drug resistance, risk factors

## Abstract

**Objectives:**

To explore the infection characteristics of different bacterial diabetic foot (DF) in Yunnan area, and to provide reference basis and clinical predictive indicators for individualized treatment of patients with diabetic foot infection (DFI) in this region.

**Methods:**

A total of 194 local permanent DF patients admitted to the Department of Endocrinology of our hospital from January 2023 to May 2024 were selected. The incidence and drug resistance of DFI were analyzed. Based on bacterial Gram staining as the reference standard, patients were divided into the single-strain Gram-positive bacterial infection group (G^+^, n=28) and the single-strain Gram-negative bacterial infection group (G^-^, n=28), and their clinical data were compared. According to the IWGDF/IDSA 2023 grading criteria, patients were divided into the DF without infection group (n=27) and the DFI group (n=167), and their clinical data were compared. Grading was independently performed by two qualified clinicians. Discrepancies were adjudicated by a third senior physician to ensure objectivity. Logistic regression was used to analyze the risk factors for DF complicated with infection, and receiver operating characteristic (ROC) curves were further drawn when statistically significant.

**Results:**

Among 194 patients, 116 underwent culture of foot ulcer tissue secretions, of which 71 had positive pathogen culture results and 45 had negative results, with a bacterial detection rate of 61.20%. The most common pathogen of bacterial diabetic foot in Yunnan area was Staphylococcus aureus, followed by Escherichia coli. Staphylococcus aureus showed high resistance to penicillin, clindamycin, and erythromycin; Escherichia coli showed high resistance to ampicillin, cefazolin, doxycycline, and compound sulfamethoxazole. The course of diabetic foot in the G^+^ group was longer than that in the G^-^ group, and the number of smokers in the G^+^ group was higher than that in the G^-^ group, with statistically significant differences (*P* < 0.05). Logistic regression analysis showed that male gender, fibrin degradation product (FDP), D-dimer, prothrombin time (PT), fibrinogen (FIB), and γ-glutamyl transpeptidase (γ-GT) were independent risk factors for DF complicated with infection. ROC curve analysis showed that the combination of D-dimer + FDP + FIB had the largest area under the curve (AUC) (0.7848, 95% CI 0.7031-0.8666) for predicting DF complicated with infection, with a sensitivity of 57.58% and a specificity of 92.31%. Conclusion The combination of D-dimer + FDP + FIB can be used as an auxiliary exclusion tool for early DFI in Yunnan area, with high specificity (92.31%) and certain clinical practical value.

## 1 Introduction

Diabetic Foot (DF), one of the most severe complications of diabetes mellitus (DM), often leads to repeated hospitalization or even amputation due to uncontrollable infection and irreparable tissue necrosis. The treatment cost is enormous, accounting for approximately one-third of the total medical expenses of DM, making it a major public health issue that imposes a heavy burden on society (1). The prevalence of DF varies significantly across countries/regions, ranging from 1.5% to 16.6%, and the annual incidence of DF in patients over 50 years old in China is 8.1% (1).Overall, the proportion of Gram-positive bacteria and Gram-negative bacteria in DFI is comparable in China, but there may be significant differences in DFI bacteria among different regions (2). Existing studies have mostly focused on central, eastern, and coastal areas of China, with scarce data on southwest ethnic minority areas. Early screening and correction of DF risk factors, as well as standardized treatment, can significantly reduce the amputation rate and medical costs, and improve patients’ quality of life. Yunnan is located in the southwest border of China, with characteristics such as multi-ethnicity, borderland, mountainous areas, and uneven distribution of primary medical resources. The early screening and intervention capabilities for DM patients are limited, leading to a high rate of delayed treatment for DFI patients. Differences in dietary structure, lifestyle, and antimicrobial use patterns among different ethnic groups result in variations in DFI pathogen spectra across different geographical regions of China (3).This means that clinicians need to refer to local epidemiological data when selecting empirical antibiotics. Therefore, this study aims to provide more targeted clinical diagnosis and treatment strategies for DFI patients in this region by analyzing the infection characteristics of different bacterial DF in Yunnan area. Developing localized empirical anti-infection regimens based on regional bacterial spectrum differences is particularly crucial.

## 2 Objects and methods

### 2.1 Study objects

A total of 194 local permanent DF patients (residing in Yunnan for ≥5 years) admitted to the Department of Endocrinology of our hospital from January 2023 to May 2024 were selected, including 142 males (142/194, 73.2%) and 52 females (52/194, 26.8%), with an average age of 61.31 ± 11.77 years. DF was diagnosed according to the Clinical Pathway for Diagnosis and Treatment of Diabetic Foot in China (2023 Edition) (4).Exclusion criteria: history of trauma within one month, combined with severe damage to other organs, infectious diseases, immune diseases, and malignant tumors, and receiving antibiotic treatment within one week. This was a single-center retrospective study, and all study objects were inpatients, which may have selection bias. Caution should be exercised when extrapolating the results to outpatients or patients in primary medical institutions. The sample size was determined based on convenience sampling without prior power analysis, which may affect the efficiency of statistical tests. This study was approved by the hospital ethics committee [(2023) Lun Shen L No. 30], and all study objects signed informed consent forms.

### 2.2 Study methods

General data of patients were collected through the hospital’s HIS system, including gender, age, height, weight, DM course, DF course, smoking status, drinking status, and past medical history, and BMI was calculated. Laboratory data of patients were collected, including routine blood tests, coagulation function, liver function, renal function, blood lipids, glucose metabolism, active vitamin D, serum calcium, and inflammatory markers. Microbial culture results of foot ulcers were collected for the first time upon admission (before antibiotic use). Before specimen collection, standardized surgical debridement was performed to remove all necrotic and devitalized tissue. All specimens were collected from the deep base of the ulcer using sterile swabs to avoid surface contamination. The samples were immediately placed in sterile culture tubes and sent for inspection. The culture time was 48–72 hours, and negative specimens were extended to 5–7 days. The type, quantity, and drug sensitivity results of cultured microorganisms were recorded. For polymicrobial infections, all detected pathogens were recorded, and the dominant flora was determined according to colony counts.

### 2.3 Statistical analysis

SPSS 27.0 software was used for statistical analysis. Normally distributed measurement data were expressed as mean ± standard deviation, and independent samples t-test was used for comparison between two groups. Count data were expressed as n (%), and χ²test was used. Logistic regression was used to analyze the risk factors for DF complicated with infection. GraphPad Prism was used to draw ROC curves, and AUC, optimal diagnostic sensitivity, specificity, and cut-off value were calculated. The sample size of this study was determined based on the actual number of local DF patients admitted to our hospital during the study period. Combined with sample size estimation of similar regional studies at home and abroad, it can meet the statistical requirements of Logistic regression and ROC curve analysis. Due to multiple comparisons, Bonferroni method was used to correct the significance level, and *P* < 0.05 after correction was considered statistically significant.

## 3 Results

### 3.1 Clinical characteristics of diabetic foot

#### 3.1.1 Incidence of diabetic foot infection

A total of 194 local permanent DF patients in Yunnan were included, including 142 males (73.2%) and 52 females (26.8%), with an average age of 61.31 ± 11.77 years. Among them, 100 cases were complicated with hypertension, 26 with coronary heart disease, 67 with fatty liver, 168 with diabetic peripheral neuropathy (DPN), and 99 with diabetic kidney disease (DKD). Among 194 patients, 116 completed foot ulcer tissue secretion culture, of which 71 had positive pathogen culture results and 45 had negative results, with a bacterial detection rate of 61.20%.

Among 71 patients with positive culture, 11 patients (15.49%) had infections with two or more microorganisms. A total of 91 strains of pathogens were detected, including 40 strains of Gram-positive bacteria (43.95%), 47 strains of Gram-negative bacteria (51.65%), and 4 strains of fungi (4.40%).

Gram-positive bacteria in the flora distribution of bacterial diabetic foot included: 27 strains of Staphylococcus aureus (accounting for approximately 29.67% of the total positive cultures, 27/91), 4 strains of Streptococcus agalactiae (4.40%, 4/91), 3 strains of Enterococcus faecalis (3.30%, 3/91), 2 strains of Corynebacterium striatum (2.20%, 2/91), 2 strains of Staphylococcus lugdunensis (2.20%, 2/91), 1 strain of Staphylococcus saprophyticus (1.10%, 1/91), and 1 strain of Streptococcus dysgalactiae (1.10%, 1/91).

Gram-negative bacteria included: 17 strains of Escherichia coli (18.68%, 17/91), 6 strains of Pseudomonas aeruginosa (6.59%, 6/91), 5 strains of Proteus mirabilis (5.49%, 5/91), 4 strains of Klebsiella pneumoniae (4.40%, 4/91), 2 strains each of Proteus hauseri, Hafnia alvei, Enterobacter cloacae, and Enterobacter hormaechei (each accounting for 2.20%, 2/91), and 1 strain each of Citrobacter braakii, Acinetobacter lwoffii, Citrobacter freundii, Morganella morganii, Proteus vulgaris, Enterobacter xiangfangensis, and Pseudomonas putida (each accounting for 1.10%, 1/91).

**Figure 1:**
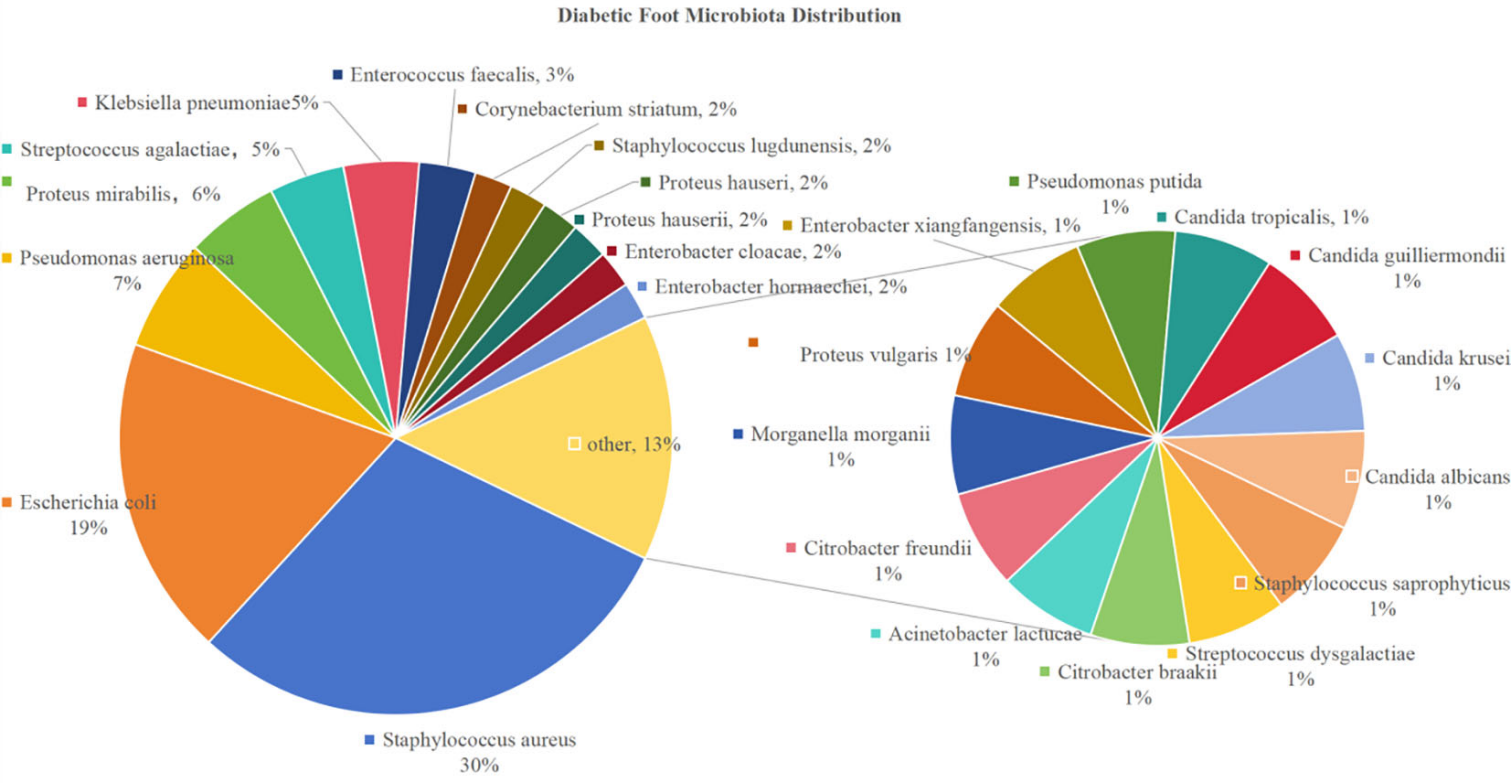
The distribution of the microbiota in diabetic foot.

Fungi were rarely detected in this study, with 4 strains including Candida tropicalis, Candida guilliermondii, Candida krusei, and Candida albicans (1 strain each, 1.10% each) (**Figure 1**).

#### 3.1.2 Distribution of bacterial resistance in diabetic foot

After analyzing the drug resistance of positive bacterial flora cultured from diabetic foot ulcer secretions, the following conclusions were drawn.

Staphylococcus aureus was highly sensitive to teicoplanin (glycopeptides), vancomycin (glycopeptides), linezolid (oxazolidinones), tigecycline (tetracyclines), daptomycin (lipopeptides), and ceftaroline (fifth-generation cephalosporins), but showed high resistance to penicillin (penicillins), clindamycin (lincosamides), and erythromycin (macrolides). Among the 21 Staphylococcus aureus isolates in the G^+^ group (out of 27 total positive cultures), 5 (23.81%) were identified as methicillin-resistant Staphylococcus aureus (MRSA) based on oxacillin resistance (**Table 1**).

**Table 1:** Antibiotic susceptibility of Staphylococcus aureus.

Antibiotic class	Antibiotic name	Susceptibility (%)
Glycopeptides	Teicoplanin	100.00
Glycopeptides	Vancomycin	100.00
Oxazolidinones	Linezolid	100.00
Tetracyclines	Tigecycline	100.00
Lipopeptides	Daptomycin	100.00
Cephalosporins	Ceftaroline(5th generation)	100.00
Rifamycins	Rifampin	96.03
Aminoglycosides	Gentamicin	88.89
Sulfonamide s	Trimethoprim-Sulfamethoxazole	77.78
Fluoroquinolones	Levofloxacin	77.78
Fluoroquinolones	Moxifloxacin	74.07
Fluoroquinolones	Ciprofloxacin	62.96
Lincosamides	Clindamycin	29.62
Macrolides	Erythromycin	33.33
Penicillins	Oxacillin	74.07
Penicillins	Penicillin G	0
Methicillin resistance	MRSA proportion	23.81 (5/21)

Escherichia coli was highly sensitive to imipenem (carbapenems), meropenem (carbapenems), colistin (polypeptides), amikacin (aminoglycosides), and tigecycline (tetracyclines), but showed high resistance to ampicillin (penicillins), cefazolin (first-generation cephalosporins), doxycycline (tetracyclines), and trimethoprim-sulfamethoxazole (sulfonamides) (**Table 2**).

**Table 2:** Antibiotic susceptibility of Escherichia coli.

Antibiotic class	Antibiotic name	Susceptibility (%)
Carbapenems	Imipenem	100.00
Carbapenems	Meropenem	100.00
Polypeptides	Colistin	100.00
Aminoglycosides	Amikacin	100.00
Aminoglycosides	Tobramycin	68.75
Aminoglycosides	Gentamicin	68.75
Tetracyclines	Tigecycline	100
Tetracyclines	Minocycline	75.00
Tetracyclines	Doxycycline	12.50
Cephalosporins	Cefoperazone-Sulbactam(3rd generation)	87.5
Cephalosporins	Ceftazidime(3rd generation)	56.25
Cephalosporins	Cefepime(4th generation)	62.50
Cephalosporins	Cefuroxime(2nd generation)	37.50
Cephalosporins	Ceftriaxone(3rd generation)	43.75
Cephalosporins	Cefazolin(1st generation)	12.50
Penicillins + β-lactamase inhibitors	Piperacillin-Tazobactam	75.00
Penicillins + β-lactamase inhibitors	Ampicillin-Sulbactam	50.00
Penicillins	Ampicillin	6.25
Monobactams	Aztreonam	62.5
Quinolones	Ciprofloxacin	43.75
Quinolones	Levofloxacin	37.5
Sulfonamides	Trimethoprim-Sulfamethoxazole	31.25

### 3.2 Clinical characteristics of diabetic foot infection caused by different bacteria

#### 3.2.1 Comparison of general data between two groups

According to the positive results of secretion culture, patients were divided into G^+^ and G^-^ groups based on bacterial Gram staining. To reduce confounding factors, only patients with single bacterial infection were included (28 cases in G^+^ group, 28 cases in G^-^ group). The course of diabetic foot in the G^-^ group was longer than that in the G^+^ group; the number of smokers in the G^+^ group was higher than that in the G^-^ group, with statistically significant differences (*P* < 0.05) (**Table 3**).

**Table 3:** Comparison of general data between the two groups [Mean ± SD; M(25%, 75%)].

Indicator	G^+^group (n=28)	G^-^group (n=28)	X^2^/t/Z	*P*
Gender (Male/Female)	23 (82.14%)/5 (17.86%)	20 (71.4%)/8 (28.6%)	0.902	0.342
Age (Years)	61.39 ± 9.85	62.46 ± 12.07	-0.364	0.717
DM duration (Years)	13.04 ± 7.22	12.32 ± 6.73	0.383	0.703
DF duration (months)	0.61 (0.25, 1.00)	1.00 (0.54, 5.50)	-2.431	0.015*
Height (cm)	166.11 ± 6.75	166.39 ± 7.86	-0.146	0.885
Weight (kg)	62.70 ± 7.99	64.75 ± 11.54	-0.774	0.443
BMI (kg/m^2^)	22.80 ± 3.16	23.41 ± 3.60	-0.682	0.498
Smoking (Yes/No) (%)	14 (50%)/14 (50%)	6 (21.43%)/22 (78.57%)	4.978	0.026*
Alcohol consumption (Yes/No) (%)	5 (17.86%)/23 (82.14%)	1 (3.57%)/27 (96.43%)	2.987	0.084
Hypertension (Yes/No)	17 (60.71%)/11 (39.29%)	13 (46.43%)/15 (53.57%)	1.149	0.284
Coronary heart disease (Yes/No) (%)	3 (10.71%)/25 (89.29%)	1 (3.57%)/27 (96.43%)	0.269	0.604
Fatty liver (Yes/No) (%)	8 (28.57%)/20 (71.43%)	8 (28.57%)/20 (71.43%)	0	1
DPN (Yes/No) (%)	24 (85.71%)/4 (14.29%)	23 (82.14%)/5 (17.86%)	0	1
DKD (Yes/No) (%)	14 (50%)/14 (50%)	15 (53.57%)/13 (46.43%)	0.072	0.789

*P < 0.05. Bold values indicate statistically significant differences.

There were no statistically significant differences in the severity grade of diabetic foot infection, the severity grade of lower extremity vascular lesions, or the location of diabetic foot ulcers between the two groups (*P*>0.05) (**Tables 4** ,**5**).

**Table 4:** Comparison of infection severity and degree of vascular lesions between the two groups.

Indicator	Group	N	Mean rank	Z	*P*
DFI Infection Classification	G^+^	28	25.64	-1.521	0.128
	G^-^	28	31.36		
Degree of Vascular Lesion	G^+^	28	28.00	-0.245	0.806
	G^-^	28	29.00		

**Table 5:** Comparison of ulcer sites.

Site Group	G^+^group	G^-^ group	X^2^	*P*
Toes	19	16	4.745	0.477
Sole of the foot	3	4		
Ankle	0	3		
Heel	0	1		
Dorsum of the foot	5	3		
Other parts	1	1		

#### 3.2.2 Comparison of laboratory data between two groups

There were no statistically significant differences in laboratory data between the two groups (*P*>0.05) (**Table 6**).

**Table 6:** Comparison of laboratory data between the two groups [Mean ± SD; M(25%, 75%)].

Indicator	G^+^group(n=28)	G^-^ group(n=28)	Z/t/t’	*P*
Absolute white blood cell count(10^9/L)	8.27(7.22, 10.33)	9.45(7.38, 14.22)	-1.123	0.262
Absolute neutrophil count(10^9/L)	5.66(4.45, 7.43)	7.18(4.68, 11.37)	-1.401	0.161
Absolute lymphocyte count(10^9/L)	1.65(1.28, 2.00)	1.37(0.96, 2.04)	-1.246	0.213
Absolute monocyte count(10^9/L)	0.51(0.43, 0.63)	0.53(0.44, 0.78)	-0.32	0.749
Hemoglobin (g/L)	127.07 ± 22.71	118.82 ± 26.30	1.256	0.214
Platelets (10^9/L)	278.50(190.00, 371.00)	331.00(269.50, 387.75)	-1.519	0.135
FDP (mg/L)	3.55(2.6, 4.60)	5.3(2.95, 7.75)	-1.754	0.079
D-dimer (mg/L)	0.77(0.48, 1.64)	0.84(0.69, 1.98)	-0.926	0.354
PT(s)	13.30(12.73, 13.68)	13.25(12.53, 14.20)	-0.507	0.615
TT(s)	18.25(17.00, 19.03)	18.20(17.73, 19.95)	-1.336	0.181
FIB(g/L)	5.82 ± 1.96	6.13 ± 2.07	-0.58	0.564
APTT(s)	37.84 ± 3.98	38.88 ± 5.91	-0.769	0.445
ALT(IU/L)	14.25(7.63, 24.10)	18.30(6.73, 27.83)	-0.623	0.533
AST(IU/L)	16.50(14.95, 21.43)	19.55(14.83, 25.08)	-1.336	0.182
Total Bilirubin (umol/L)	8.55(6.48, 11.45)	8.00(6.08, 14.05)	-0.197	0.844
Albumin (g/L)	34.15(31.18, 37.83)	31.80(28.73, 39.68)	1.151	0.256
Globulin (g/L)	39.39 ± 8.05	39.03 ± 6.97	0.181	0.857
Albumin/Globulin Ratio	0.80(0.7, 1.1)	0.90(0.7, 1.1)	-0.19	0.849
ALP(IU/L)	90.80(77.83, 110.38)	92.30(72.95, 137.33)	-0.549	0.583
γ-GT(IU/L)	30.50(19.50, 47.75)	40.00(16.75, 77.70)	-0.443	0.658
Cholinesterase (KU/L)	5.90(4.93, 6.98)	5.60(3.68, 7.28)	0.604	0.549
Total Bile Acids (umol/L)	2.80(1.68, 5.88)	3.25(2.23, 4.08)	-0.115	0.909
Urea (mmol/L)	6.17(4.25, 8.07)	7.21(4.12, 8.59)	-0.746	0.456
Creatinine (umol/L)	72.45(63.13, 101.05)	85.10(63.55, 115.11)	-1.065	0.287
Uric Acid (umol/L)	297.70(247.08, 394.40)	292.90(219.55, 363.95)	-0.737	0.461
Triglyceride (mmol/L)	1.10(0.92, 1.72)	1.22(0.88, 1.91)	-0.574	0.566
Total Cholesterol (mmol/L)	3.74(3.31, 4.24)	3.50(3.15, 4.23)	-0.475	0.635
LDL (mmol/L)	2.13(1.88, 2.60)	1.94(1.57, 2.69)	-0.975	0.329
HDL (mmol/L)	0.87(0.71, 1.16)	0.93(0.71, 1.14)	-0.115	0.909
Fructosamine (mmol/L)	392.00(324.05, 473.23)	363.95(308.58, 434.78)	-1.032	0.302
HbA1c (%)	10.35(8.13, 12.30)	9.30(7.83, 13.63)	-0.164	0.870
Fasting Blood Glucose (mmol/L)	8.82(4.33, 10.86)	6.30(4.98, 9.39)	-1.082	0.279
Fasting Insulin (mU/L)	11.76(8.07, 15.70)	15.75(6.79, 21.65)	-0.606	0.544
Fasting C-peptide (ng/mL)	0.81(0.55, 1.48)	1.13(0.46, 2.05)	-0.761	0.447
CRP(ng/mL)	31.85(10.58, 78.95)	39.45(4.55, 110.88)	-0.180	0.857
Active Vitamin D (nmol/L)	38.46(29.17, 48.52)	30.60(20.08, 44.57)	-1.524	0.127

### 3.3 Risk factor analysis for infection in DF patients

#### 3.3.1 Comparison of general and laboratory data between infected and non-infected groups

Referring to the IWGDF/IDSA Guidelines on the Diagnosis and Treatment of Diabetes-related Foot Infections (2023) (5), 194 patients were divided into two groups: DFI group (IWGDF/IDSA grade 2-4, n=167) and non-infected DF group (IWGDF/IDSA grade 1, n=27). There were statistically significant differences between the two groups in gender, diastolic blood pressure, absolute white blood cell count, absolute neutrophil count, absolute monocyte count, FDP, D-dimer, PT, FIB, APTT, albumin, globulin, albumin/globulin ratio, γ-GT, cholinesterase, urea, uric acid, triglyceride, HbA1c, random blood glucose, fasting C-peptide, and serum calcium (*P* < 0.05) (**Table 7**).

**Table 7:** Comparison of general and laboratory data between the two groups [M(25%, 75%)].

Indicator	the DFI group(n=167)	the uninfected DF group(n=27)	X^2^/Z/t’	*P*
Gender (Male/Female)	127(76.00%)/40(24.00%)	15(55.60%)/12(44.40%)	4.975	0.026
Diastolic Blood Pressure (mmHg)	77(69, 84)	81(77, 93)	-2.44	0.015
Absolute white blood cell count(10^9/L)	8.05(6.46, 11.38)	6.32(5.61, 7.42)	-4.189	<0.001
Absolute neutrophil count(10^9/L)	5.66(4.38, 9.05)	4.10(3.14, 5.30)	-3.994	<0.001
Absolute monocyte count(10^9/L)	0.49(0.36, 0.64)	0.35(0.31, 0.43)	-3.741	<0.001
FDP (mg/L)	3.40(2.20, 5.80)	1.95(1.50, 2.80)	-4.047	<0.001
D-dimer (mg/L)	0.72(0.365, 1.565)	0.315(0.20, 0.4325)	-4.502	<0.001
PT(s)	13.00(12.50, 13.70)	12.50(12.10, 12.95)	-2.752	0.006
FIB(g/L)	5.27(4.21, 6.91)	4.11(3.4175, 4.88)	-3.771	<0.001
APTT(s)	37.20(34.90, 40.00)	34.75(31.925, 38.675)	-2.440	0.015
Albumin (g/L)	34.90(30.20, 39.40)	39.70(35.50, 41.50)	-2.995	0.003
Globulin (g/L)	36.20(31.70, 42.80)	31.40(26.70, 34.80)	-3.902	<0.001
Albumin/Globulin Ratio	1.00(0.70, 1.20)	1.20(1.10, 1.55)	-4.340	<0.001
γ-GT(IU/L)	34.00(20.00, 64.00)	25.75(14.25, 43.75)	-2.364	0.018
Cholinesterase (KU/L)	6.30(4.80, 7.90)	8.50(7.425, 9.750)	5.732	<0.001
Urea (mmol/L)	6.00(4.23, 8.28)	7.80(5.37, 10.10)	-2.106	0.035
Uric Acid (umol/L)	297.00(231.80, 377.10)	384.65(323.525, 489.325)	-3.343	<0.001
Triglyceride (mmol/L)	1.20(0.92, 1.79)	1.85(1.03, 2.315)	-2.034	0.042
HbA1c (%)	9.20(7.50, 11.40)	7.50(6.875, 9.550)	-2.275	0.023
Random blood glucose(mmol/L)	11.40(8.28, 17.30)	9.00(6.93, 11.90)	-2.429	0.015
Fasting C-peptide (ng/mL)	1.04(0.62, 1.71)	1.88(1.00, 2.65)	-2.81	0.005
Serum Calcium (mmol/L)	2.20(2.11, 2.28)	2.33(2.22, 2.39)	-3.838	<0.001

#### 3.3.2 Multicollinearity analysis for DFI occurrence

To further explore the influencing factors of DF infection, collinearity test was performed on indicators with statistically significant differences in baseline data comparison (**Table 8**). With VIF≥10 as the exclusion criterion, the results indicated no multicollinearity between the above indicators.

**Table 8:** Multicollinearity Test for the Progression of DF to DFI.

Indicator	VIF	Indicator	VIF
Gender	1.309	Diastolic Blood Pressure	1.421
Absolute white blood cell count	1.518	Absolute neutrophil count	1.551
Absolute monocyte count	2.110	FDP	6.585
D-dimer	7.890	PT	1.631
FIB	2.456	APTT	1.347
Albumin	4.627	Globulin	1.871
Albumin/Globulin Ratio	3.669	γ-GT	1.728
Cholinesterase	2.866	Urea	1.866
Uric Acid	2.077	Triglyceride	1.635
HbA1c	2.267	Random blood glucose	1.835
Fasting C-peptide	1.311	Serum Calcium	2.465

#### 3.3.3 Binary logistic regression analysis of infection in DF

Although there was no multicollinearity between the above indicators, considering the causal relationship between DFI and absolute white blood cell count, absolute neutrophil count, absolute monocyte count, globulin, and albumin/globulin ratio, these indicators were excluded. The remaining indicators were included in binary Logistic regression analysis, which showed that male gender, FDP, D-dimer, PT, FIB, and γ-GT were independent risk factors for DF infection (*P* < 0.05), while diastolic blood pressure, albumin, cholinesterase, and serum calcium were protective factors (*P* < 0.05) (**Table 9**).

**Table 9:** Binary logistic regression analysis of infection in DF.

Indicator	B	SE	Wald	OR	95%CI	*P*
Male	0.932	0.428	4.752	2.540	1.099-5.873	0.029
Diastolic Blood Pressure	-0.033	0.015	4.471	0.968	0.939-0.998	0.034
FDP	0.485	0.165	8.607	1.623	1.174-2.244	0.003
D-dimer	2.020	0.695	8.458	7.541	1.932-29.428	0.004
PT	0.593	0.258	5.278	1.810	1.091-3.001	0.022
FIB	0.619	0.184	11.300	1.857	1.294-2.664	<0.001
Albumin	-0.131	0.044	9.047	0.877	0.805-0.955	0.003
γ-GT	0.022	0.011	4.166	1.023	1.001-1.045	0.041
Cholinesterase	-0.433	0.110	15.504	0.648	0.522-0.804	<0.001
Serum Calcium	-8.197	2.243	13.361	0.000	0.000-0.022	<0.001

#### 3.3.4 Efficacy analysis of risk factors for infection in DF

**Figure 2:**
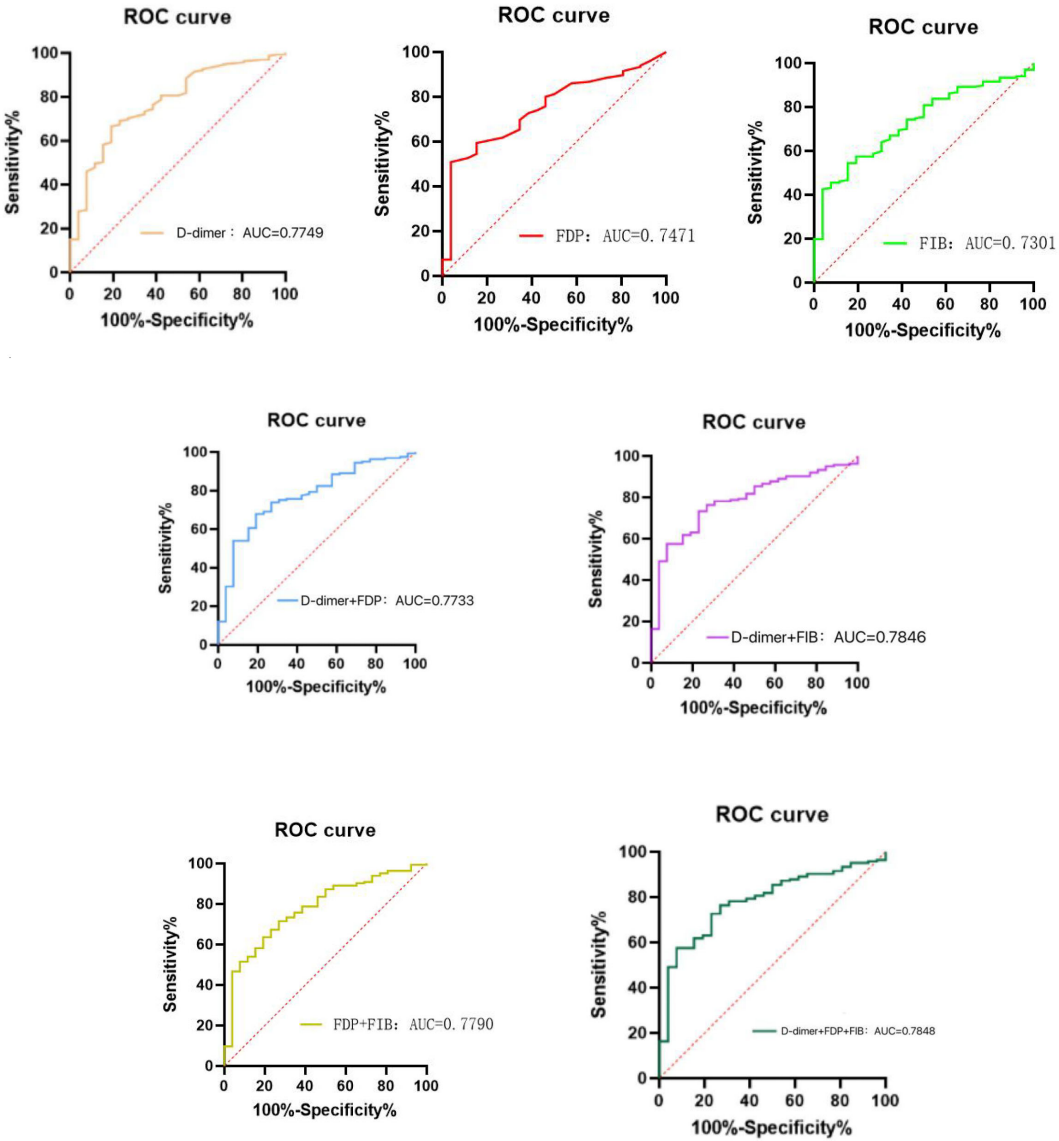
(1) ROC curve of D-dimer for predicting infection in patients with DF. (2) ROC curve of FDP for predicting infection in patients with DF. (3) ROC curve of FIB for predicting infection in patients with DF. (4) ROC curve of D-dimer +FDP for predicting infection in patients with DF. (5) ROC curve of D-dimer +FIB for predicting infection in patients with DF. (6) ROC curve of FDP +FIB for predicting infection in patients with DF. (7) ROC curve of D-dimer +FDP +FIB for predicting infection in patients with DF.

To further understand the efficacy of male gender, FDP, D-dimer, PT, FIB, and γ-GT in predicting DF infection, ROC curve analysis was performed using GraphPad Prism. An AUC >0.7 indicates strong predictive efficacy. The results showed that the combination of D-dimer + FDP + FIB had the highest AUC of 0.7848, indicating moderate to high predictive efficacy. However, the sensitivity of this combined indicator was only 57.58%, meaning nearly half of infected patients may be missed, so it has limited value as a screening tool. The high specificity of 92.31% indicates that when the indicator is negative (i.e., below the optimal cut-off value of 0.8868), it has high value in excluding infection and can be used as an auxiliary exclusion tool (**Table 10**) (**Figure 2**).

**Table 10:** ROC curve analysis of infection in patients with DF.

Indicator	*AUC*	*95%CI*	Youden index	Sensitivity (%)	Specificity (%)	Optimal cut - off value
D-dimer	0.7749	0.6824-0.8675	0.4744	66.67	80.77	≥0.475
FDP	0.7471	0.6584-0.8357	0.4721	50.91	96.15	≥3.300
FIB	0.7301	0.6414-0.8188	0.3911	54.49	84.62	≥4.995
D-dimer+FDP	0.7733	0.6831-0.8635	0.4865	67.88	80.77	≥0.8139
D-dimer+FIB	0.7846	0.7029-0.8664	0.5025	73.33	76.92	≥0.8157
FDP+FIB	0.7790	0.6906-0.8675	0.4460	71.52	73.08	≥0.8299
D-dimer+FDP+FIB	0.7848	0.7031-0.8666	0.4989	57.58	92.31	≥0.8868

## 4 Discussion

Diabetic Foot Ulcer (DFU), one of the most severe complications of diabetes mellitus, poses an undeniable global health challenge. The global prevalence of DFU among diabetic patients is approximately 6.3% (6, 7).However, regional studies have reported even higher rates, such as 10%-30% in African countries (excluding North Africa) (8).This elevated prevalence in Africa has been attributed to the prevalent practice of barefoot walking in local rural areas (6).Additionally, diabetic patients face a 19%-34% lifetime risk of developing foot ulcers (7, 9), underscoring the long-term health threat posed by DFU. Such a high prevalence and lifetime risk, coupled with DFU being the leading cause of amputation and disability in diabetic patients, necessitate long-term medical intervention for a large number of patients—resulting in substantial healthcare expenditures. The direct medical costs associated with diabetic foot complications, particularly DFU, are extremely high, with studies indicating they are comparable to those of cancer treatment. For instance, the direct medical cost of diabetes in the United States reached $237 billion in 2017, approximately one-third of which was attributed to the diagnosis and treatment of diabetic foot. Based on this calculation, the direct costs related to diabetic foot are close to the $80.2 billion in direct cancer costs in the United States in 2015 (10).Addressing this challenge requires strengthening preventive measures and promoting early intervention technologies to reduce the incidence and severity of diabetic foot, thereby alleviating the associated economic burden.

Bacteria play a pivotal role in the occurrence, progression, and deterioration of DFU. These ulcerated wounds serve as “breeding grounds” for bacterial colonization, infection, and dissemination, which further exacerbate disease complexity and profoundly impact patient treatment and prognosis. Through the isolation and identification of bacteria from DFU wounds in Yunnan, this study revealed a unique pathogen spectrum: Gram-negative bacteria (51.65%) were slightly more prevalent than Gram-positive bacteria (43.95%). This distribution aligns with the trend of Gram-negative bacteria predominance in DFU patients in developing Asian countries such as India (11), and is consistent with the macro rule proposed by Macdonald et al. (12)that Gram-negative bacteria are the main cause of DFI in developing countries. This further confirms the correlation between DFU wound bacterial spectra and regional economic development levels. As a multi-ethnic province in southwest China, Yunnan exhibits characteristics such as diverse dietary structures, lifestyles, and antimicrobial use patterns among different ethnic groups, which may collectively shape the unique local pathogen spectrum. Combined with the “national income-related bacterial spectrum distribution rule” proposed by Macdonald et al. (12), this difference in bacterial spectra may stem from multiple overlapping factors: developed countries have more abundant medical resources, enabling timely early intervention for DFU patients, such that wound infections are often in the early stage—where Gram-positive bacteria (e.g., Staphylococcus aureus), common skin and mucosal commensals, tend to dominate. In contrast, patients in developing countries may experience delayed medical consultation and limited hygiene conditions, leading to more complex wound infections. Gram-negative bacteria, with their stronger environmental adaptability and biofilm-forming capacity (13, 14), are more likely to proliferate and become dominant flora in chronic wounds. Additionally, this study found that the proportion of smokers in the Gram-positive bacterial infection group was significantly higher than that in the Gram-negative bacteria infection group (*P* < 0.05), while the course of diabetic foot in the Gram-negative bacteria infection group was significantly longer than that in the Gram-positive bacterial infection group (*P* < 0.05). Literature review indicates that smoking may increase susceptibility to Gram-positive bacterial infections by reshaping the microbiota (15, 16), and impairing host immunity (17).Chronic wounds with a long course are more prone to biofilm formation, providing favorable conditions for the persistence of Gram-negative bacteria (13, 14).Specifically, the high detection rates of Staphylococcus aureus (29.67%) as the main Gram-positive pathogen and Escherichia coli (18.68%) as the main Gram-negative pathogen in this study reflect the universal pathogenicity of these two bacteria in DFU infections, consistent with global multi-regional epidemiological reports (18–21).

In summary, from the perspective of the association between regional characteristics and global commonalities, the slight predominance of Gram-negative bacteria in Yunnan aligns with findings from developing countries such as India, confirming the impact of economic development level and medical accessibility on bacterial spectra. Meanwhile, the cross-regional high detection rates of Staphylococcus aureus and Escherichia coli reveal a “pathogen commonality amid regional differences” in DFI—regardless of economic level, skin commensals and intestinal-derived Gram-negative bacteria are core participants in wound infections. This commonality provides a reference for formulating DFU anti-infection strategies, emphasizing that these two bacteria should be prioritized for empirical antimicrobial coverage. In clinical practice, certain patient characteristics can provide important clues for empirical anti-infection treatment. This study found that DF patients with a smoking history in Yunnan had a higher proportion of Gram-positive bacterial infections (*P* < 0.05), suggesting that initial treatment for smokers should specifically focus on covering Gram-positive cocci (especially Staphylococcus aureus). In contrast, DF patients with a longer disease course were more prone to Gram-negative bacterial infections (*P* < 0.05), requiring enhanced vigilance against Gram-negative bacilli (especially Escherichia coli) and attention to wound biofilm clearance. Based on this, combined with local pathogen spectra and drug resistance characteristics, empirical anti-infection regimens for DFI patients in Yunnan should cover both Gram-positive and Gram-negative bacteria before obtaining drug sensitivity results. Given the high resistance of Staphylococcus aureus to penicillin, clindamycin, and erythromycin, and the high resistance of Escherichia coli to ampicillin and cefazolin, these drugs are not recommended as first-line empirical treatment options. For mild infections, oral agents with both Gram-positive and Gram-negative activity (e.g., levofloxacin) or broad-spectrum β-lactam antibiotics may be considered. For moderate to severe infections, β-lactamase inhibitor combinations (e.g., piperacillin-tazobactam) combined with anti-Gram-positive agents, or carbapenems based on disease severity and drug resistance risk, are recommended. Given the MRSA proportion of 23.81% among Staphylococcus aureus isolates in this study, empirical coverage for MRSA should be considered in patients with severe infections or those with risk factors for multidrug-resistant organisms. If MRSA infection is highly suspected clinically, escalation to linezolid or vancomycin is required. Additionally, the polymicrobial infection rate in this study reached 15.49% (11/71), predominantly involving mixed Gram-positive and Gram-negative bacteria. Therefore, for patients with poor initial treatment response or severe infections, polymicrobial infection should be fully considered, and regimens adjusted promptly based on clinical response. After obtaining drug sensitivity results, targeted treatment should be implemented as soon as possible to optimize antimicrobial use and reduce the emergence of drug-resistant bacteria. Continuous monitoring of local bacterial resistance spectra and dynamic adjustment of anti-infection strategies are necessary to balance treatment efficacy and drug resistance risks.

Coagulopathy plays a critical role in the progression of DFI, characterized by an imbalance between the coagulation and fibrinolytic systems that triggers a cascade reaction throughout the entire course of infection. The D-dimer + FIB + FDP combined detection scheme explored in this study accurately captures this complex imbalance, and its significant predictive value for DF infection in Yunnan has been validated by data: an AUC of 0.7848 indicates moderate to high discriminatory power for infection risk. A high specificity of 92.31% means that when the test result is negative (i.e., below the optimal cut-off value of 0.8868), it has high value in excluding infection, which is crucial for avoiding over-medicalization and antimicrobial abuse. However, a relatively low sensitivity of 57.58% indicates a high risk of missed diagnosis when used as a screening tool, such that it is more suitable as an auxiliary exclusion tool rather than an independent diagnostic or screening tool. In clinical practice, priority should be given to reducing the risk of false-negative results because missed DFI may lead to adverse outcomes such as amputation. Therefore, a threshold with higher sensitivity may be more appropriate in clinical decision-making. In clinical settings, patients with a positive combined test result (>0.8868) should be highly suspected of infection, requiring comprehensive judgment based on clinical symptoms, other inflammatory markers, and etiological examinations. For patients with a negative test result, it provides strong support for ruling out infection. Mechanistically, the three indicators reveal the pathological essence of DF infection from different dimensions: elevated D-dimer levels not only reflect microthrombosis and tissue ischemia but are also closely associated with chronic diabetes-related inflammation, microvascular injury, and neuropathy (22–26). This study confirms that D-dimer is an independent risk factor for DF infection, consistent with previous findings that “elevated D-dimer is associated with multidrug-resistant microbial infections in DFU (27)” and “elevated D-dimer can predict poor prognosis in DFI patients (28)”. This suggests that elevated D-dimer may exacerbate local hypoxic-ischemic microenvironments, impair tissue anti-infection capacity, and create conditions for bacterial colonization. As a key mediator of the coagulation cascade, FIB is significantly elevated in type 2 diabetic patients due to glucose metabolism disorders and insulin resistance (29, 30). It participates in DF progression through pro-inflammatory responses (e.g., activating inflammatory cells to release pro-inflammatory factors) and endothelial injury (31, 32), and is positively correlated with Wagner grading (33)—higher grades (indicating more extensive ulcer depth and infection) are associated with higher FIB levels. The identification of FIB as an independent risk factor for infection in this study further confirms its pathophysiological role in promoting infection by exacerbating microcirculatory disorders and impairing foot perfusion, providing evidence for “identifying high-risk DFU patients through FIB levels (31, 34)”. As a marker of fibrinolytic activity, elevated FDP promotes DF infection through three mechanisms: 1) exacerbating local inflammation by affecting leukocyte migration and cytokine production (35); 2) inducing increased vascular endothelial permeability to aggravate microcirculatory disorders (36); 3) impairing platelet function, preventing the formation of stable thrombi at wound sites, and prolonging ulcer exposure time to facilitate bacterial colonization (37). Additionally, diabetes-induced microenvironmental changes inhibit the positive role of FDP in cell signaling, hindering cell migration and proliferation and further delaying wound healing (38). The conclusion that FDP is an independent risk factor in this study reflects the combined effect of these mechanisms.

The superior predictive efficacy of the combined three indicators in this study stems from their synergistic effect: D-dimer focuses on end products of coagulation activation, FIB reflects pro-inflammatory responses and endothelial injury in the initial stage of coagulation, and FDP reveals the end state of fibrinolytic system imbalance. Together, they construct a comprehensive pathological panorama of DF infection from different links of the coagulation-fibrinolytic axis (29, 39, 40), resulting in predictive efficacy far exceeding that of individual indicators. This study also identified diastolic blood pressure, albumin, and serum calcium as protective factors, providing systemic targets for DF infection prevention and control in Yunnan: diastolic blood pressure maintains peripheral foot perfusion pressure, ensuring tissue oxygen and nutrient delivery to reduce ischemic injury; albumin, an important nutritional indicator, not only provides raw materials for tissue repair but also supports wound healing by maintaining plasma colloid osmotic pressure and inhibiting excessive inflammatory responses—especially when albumin levels are <30g/L, the risk of infection increases significantly, highlighting the necessity of nutritional support; serum calcium indirectly reduces the risk of ulcer exposure by participating in coagulation factor activation and maintaining coagulation function stability. These findings have clear clinical implications for Yunnan: it is recommended that DF patients complete combined coagulation testing within 24 hours of admission. For high-risk populations with results >0.8868, early intensive anti-infection treatment (e.g., combined regimens covering both Gram-negative and Gram-positive bacteria) should be initiated, along with targeted microcirculation improvement (e.g., vasodilator therapy) and local thrombus clearance. For patients with hypoalbuminemia, enteral or parenteral nutritional support should be promptly provided to correct negative nitrogen balance; for patients with low diastolic blood pressure, antihypertensive regimens should be optimized to ensure foot perfusion.

This study has several limitations. First, the sample size of the G^+^and G^-^ groups was relatively small (n=28 each), which may limit statistical power. Second, as a retrospective study, 1:1 matching for confounding factors was not performed, and only logistic regression was used for adjustment. Third, formal inter-rater reliability testing (e.g., Kappa) was not performed. Fourth, the fungal detection rate was low (4.40%), and resistance profiles of other common pathogens and MDROs were not analyzed due to limited sample sizes. Fifth, the bacterial detection rate was 61.20%, and negative results may be related to prior antibiotic use or biofilm-associated bacteria. Sixth, the combined D-dimer + FDP + FIB indicator showed low sensitivity, and its combination with traditional inflammatory markers (CRP, PCT) was not explored. Seventh, the comparative analysis of pathogen spectra was limited to international studies; a detailed comparison with other regions of China was not performed. Eighth, the mechanistic discussion of the coagulation-fibrinolysis system was relatively superficial, and differences across Wagner grades were not compared due to limited sample size. Ninth, polymicrobial infections (15.49%) were excluded from the G^+^/G^-^comparison, and the optimal cutoff value was determined using the Youden index without accounting for asymmetric clinical consequences. Tenth, the study sample had a marked gender imbalance (73.2% male), which may have influenced the regression results. Therefore, the results should be interpreted with caution.

However, D-dimer, FIB, and FDP are susceptible to non-specific factors such as surgery and trauma (41), and their test results should be comprehensively interpreted in the context of clinical conditions. This study is a single-center retrospective study with a limited sample size, and the predictive efficacy of the combined three indicators has not been validated by multi-center studies. Future large-scale multi-center studies are needed to verify the stability of the combined scheme, and further explore its combined application with inflammatory markers such as CRP and procalcitonin to optimize the prediction model, achieving precise risk stratification of DF infection and providing more reliable basis for individualized treatment and prognosis evaluation. The establishment of this combined detection scheme not only provides a new tool for the early identification of DF infection in Yunnan but also deepens the understanding of DF pathophysiology from the perspective of coagulation-infection crosstalk.

## 5 Conclusion

In conclusion, this study systematically depicts the pathogen spectrum characteristics of DFI in Yunnan, revealing a unique distribution with Gram-negative bacteria slightly predominating and Staphylococcus aureus and Escherichia coli as the main pathogens. It also clarifies the clinical association between smoking, long disease course, and specific bacterial infections, providing evidence-based decision support for local empirical anti-infection treatment. Based on this, a hierarchical and individualized empirical medication approach is proposed, emphasizing the need to cover both Gram-positive and Gram-negative bacteria before obtaining drug sensitivity results, optimize selections based on clinical characteristics (smoking history, disease course), remain vigilant against polymicrobial infections, and adhere to the principle of de-escalation therapy. Additionally, this study is the first to validate the predictive value of D-dimer + FDP + FIB combined detection in Yunnan DFI patients, confirming its clinical utility as an auxiliary exclusion tool for infection, and identifying protective factors such as diastolic blood pressure, albumin, and serum calcium—providing new intervention targets for infection prevention and control. Despite limitations including a single-center design and limited sample size, as well as the need to improve the sensitivity of the combined indicators, the study results still provide important references for the early identification, precise treatment, and rational antimicrobial use of DFI in Yunnan. Future research should explore whether differences in dietary and lifestyle patterns among ethnic groups in Yunnan affect the DFI pathogen spectrum through stratified studies, providing a basis for more precise localized diagnosis and treatment. Furthermore, large-scale multi-center prospective studies are needed to verify and optimize the prediction model, explore additional novel biomarkers, and ultimately achieve full-cycle refined management of diabetic foot infection, reducing patient amputation rates and medical burdens.

## Data Availability

The original contributions presented in the study are included in the article/supplementary material. Further inquiries can be directed to the corresponding author.
